# Human mobility and socioeconomic datasets of the Rio de Janeiro metropolitan area

**DOI:** 10.1016/j.dib.2023.109695

**Published:** 2023-10-18

**Authors:** Júlio César Chaves, Moacyr A.H.B. da Silva, Ricardo de Souza Alencar, Alexandre G. Evsukoff, Vinícius da Fonseca Vieira

**Affiliations:** a EMAp/Getulio Vargas Foundation, Praia de Botafogo 190, Botafogo, 22253-900, Rio de Janeiro, Brazil; b Coppe/Federal University of Rio de Janeiro, 68506, Rio de Janeiro, Brazil; c Universidade Federal de São João del Rei, Praça Frei Orlando, 170, 36307-352, São João del Rei, Brazil

**Keywords:** Human mobility, Socioeconomic indicators, Call detail records, Mobile phone data, Rio de janeiro metropolitan area, Transportation planning

## Abstract

This data descriptor presents two main datasets and a set of auxiliary files. The mobility dataset presents a long-term study of human mobility in the Rio de Janeiro Metropolitan Area (RJMA) performed in the entire year of 2014 based on mobile phone data. The socioeconomic dataset presents selected socioeconomic variables of the Brazilian 2010 census. A set of auxiliary files is included to present georeferenced information and geographic features (shapefiles) and data used to validate the mobility estimates. The human mobility estimation was carried out using a methodology that allows direct integration with census data, based on an approximation of the geographic boundaries of census units by an aggregation of Voronoi polygons of the mobile phone antennas. The study area is the Brazilian local area 21, which includes the entire RJMA and four other municipalities. The mobility dataset is divided into two files: one is an estimation of the origin-destination (OD) matrix per day, and the other is a visitors’ dataset where the number of visitors of each location is estimated in four shifts each day. The socioeconomic dataset presents information of 55 variables for each location, which have been used in different studies and present the longest human mobility dataset available for public use.

Specification Table

**Table:** 

Subject	Transportation, Social Sciences
Specific subject area	Human mobility and socioeconomic variables
Data type	File and GIS (shapefile)
How the data were acquired	The mobility datasets were derived from a raw call detail records (CDR) dataset, which is itself undisclosed. The socioeconomic data were extracted from the 2010 Brazilian census database.
Data format	Tab delimited
Description of the data collection	This data descriptor presents two main datasets and a set of auxiliary files. The mobility dataset presents a long-term study of human mobility in the Rio de Janeiro Metropolitan Area (RJMA) performed in the entire year of 2014 based on mobile phone data. The socioeconomic dataset presents selected socioeconomic variables of the Brazilian 2010 census. A set of auxiliary files is included to present georeferenced information and geographic attributes (shapefiles) and data used to validate the mobility estimates.
Data source location	**Institutions**: Universidade Federal do Rio de Janeiro – UFRJ and Fundação Getulio Vargas - FGV **City**: Rio de Janeiro **Region**: Rio de Janeiro Metropolitan Area **Country**: Brazil
Data accessibility	**Repository name**: FGV Dataverse **Direct URL to Data:** https://dataverse.fgv.br/dataverse/RJMA

## 1 Value of the Data

• The datasets provide support to human mobility research. The mobility dataset unveils daily mobility patterns throughout the entire 2014 year, as well as changes in these patterns due to weekdays, weekends, and holidays. It shows a detailed picture of the mobility patterns in the RJMA.
• Mobility patterns can be directly linked to socioeconomic variables of the origin and/or destination locations, allowing studies on the relationship between mobility and socioeconomic variables. Recent studies have shown the relation between mobility and inequality. This dataset has been used in one of such studies [8].
• Transportation models represent a challenge for epidemiological modeling, especially in Brazil, where public mobility data with a defined scope and methodology is not widely available.

## 2 Objectives

This data descriptor presents two main datasets and a set of auxiliary files. The mobility dataset presents a long-term study of human mobility in the Rio de Janeiro Metropolitan Area (RJMA) performed in the entire year of 2014 based on mobile phone data. The socioeconomic dataset presents selected socioeconomic variables of Brazilian 2010 census. A set of auxiliary files is included to present georeferenced information and geographic features (shapefiles) and data used to validate the mobility estimates.

The study area comprises the RJMA in its geographic limits of 2013, which covers 5327 km² and has 12.7 million inhabitants (according to 2010 census) living in 19 municipalities around the city of Rio de Janeiro, which has 6.3 million inhabitants. The raw data were collected from the voice calls through 1078 antennas located in the area code 21, which includes all the RJMA and five small-medium neighboring municipalities: Tanguá, Rio Bonito, Cachoeiras de Macacu, Teresópolis and Mangaratiba. The Mangaratiba municipality has been removed because after pre-processing, the number of users with an identified domicile was much lower than other municipalities. This led us to the conclusion that there may have been sampling issues in this specific area.

The raw mobile phone dataset covers 363 days in the period from December 31st, 2013, to January 1st, 2015 (4 days were missing), totaling 2.1 billion records for 2.9 million mobile phone users.

**Fig. 1:**
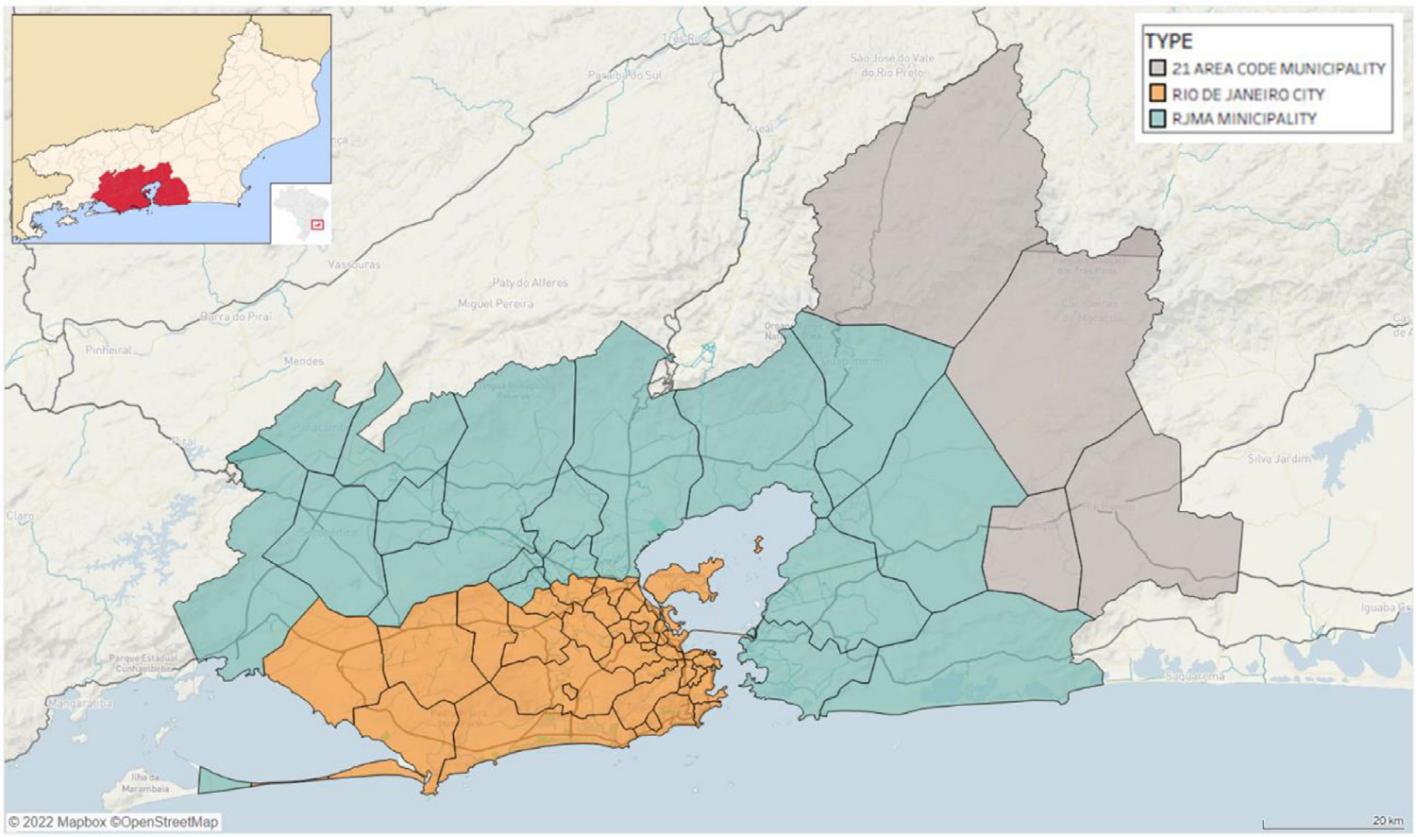
Overview of the study area.

Fig. 1 presents an overview of the study area with the adopted spatial partitioning in 54 locations or geographic units: the Rio de Janeiro city was itself partitioned in 32 sub-districts, the 17 other municipalities of the RJMA and the five municipalities that are in area code 21, but not in the RJMA on its 2013 limits^4^.

The human mobility estimation was carried out using a methodology that allows direct integration with census data, based on an approximation of geographic boundaries of census units by an aggregation of Voronoi polygons representing the coverage of the mobile phone towers.

The datasets presented in this data descriptor have been used in different studies. Barboza et al. [2] present the basic methodology used for diary origin-destination (OD) matrix estimation and the analysis of mobility patterns. Chaves et al. [4] discuss the variation of the gravity model parameters in the case of large events. Lenormand et al. [8] present a study using entropy as a measure of attractiveness that unveil socioeconomic inequalities in RJMA.

## 3 Data Description

The raw call detail records (CDR) data used to derive the data presented in this descriptor cannot be disclosed, as it may breach users' privacy. According to the Non-Disclosure Agreement (NDA) signed by the authors, the name of the carrier cannot be disclosed, but the market share in the study area was an average of 20% in 2014. The signed NDA allows authors to publish statistical results from the raw CDR data, such as the aggregated data presented in this data descriptor.

Mobile phone CDR data have been widely used can be used for mobility estimation as the user position can be referred to the nearest antenna at each record, which can be a call, a text message, or an internet access. In this study only voice call records were available. Each antenna is georeferenced by the coordinates of the tower where it is located. Generally, at least 3 antennas are positioned at 120° in each tower, so tower coverage can be approximated using Voronoi polygons.

The study area was partitioned into 54 geographic units, as shown in Fig. 1. The spatial partitioning considers the demographic and socioeconomic locations defined by the Brazilian Institute for Geography and Statistics (IBGE). The IBGE uses global standards so that the methodology can be replicated in other cities and metropolitan areas.

Each geographical unit approximates the geographical boundary of a sub-district (inside Rio de Janeiro city) or municipality (RJMA outside Rio). The geographical unit is the aggregation of the Voronoi polygons of the antennas inside the corresponding location. The result of this spatial partitioning is a set of locations that can be directly related to all the surveys carried out by the IBGE and/or other surveys using the same partitioning.

The data is organized in three datasets, described next:

• **Mobility dataset**: Contains two files with different estimations of mobility: one file is an estimation of the origin-destination (OD) matrix per day and the other file is an estimation of the number of visitors at each location in four shifts each day.
• **Socioeconomic dataset**: Contains one file extracted form Brazilian 2010 census with 55 socioeconomic variables for each one of the 54 geographic units.
• **Auxiliary files**: Contains georeferenced information and geographic features in shapefiles for the RJMA and a file with data from the 2013 Rio de Janeiro Transportation Survey, used for validation.

### 3.1 Mobility dataset

The mobility dataset presents the results of mobility estimations from the raw CDR data for each one of the geographic units. Two files of mobility estimations are available: the visitors file and the origin-destination (OD) file.

The visitors file presents as estimation of the number of visitors detected at each location in four shifts each day, computed using at least one phone call. As each user is related to a presumed domicile, it is possible to group the visitors by domicile. Additional attributes were included to inform about seasons, holidays, etc. This file contains 4,078,668 records, which is the product of 54 locations of domicile, 54 visited locations in 4 shifts, and 363 days. The number of visitors is adjusted to the population as described in Section 3. The visitors’ file is available in tab-delimited format in the FGV Dataverse^5^. Table 1 presents the data dictionary.

**Table 1:** Data dictionary of the visitors’ file.

Variable	Type	Description	Domain
day	String (12)	Date of the visit. Starting at 2013-12-31.	363 dates
shift	String (9)	Name of the shift.	{“AFTERNOON”, “MORNING”, “NIGHT”, “WORK”}
day_type	String (11)	Type of the date.	{“holiday”, “nonworkday”, “workday”}
day_description	String (33)	Description of the date.	63 descriptions of dates
season	String (9)	Name of the season.	{“AUTUMN”, “SPRING”, “SUMMER”, “WINTER”}
presumed_domicile	String (20)	Residence location name.	Name of the 54 geographic units
destination	String (20)	Visited location name.	Name of the 54 geographic units
census_population	Long integer	Population of residence location.	Natural numbers.
cell_phone_users	Long integer	Number of users detected in the visited location.	Natural numbers.
total_detected _users	Long integer	Number of users from residence location detected at visited location.	Natural numbers.
visiting_population	Long integer	Number of visitors from residence location detected at visited location.	Natural numbers.
estimated_distance _travelled	Long integer	Distance (in meters) from residence location to visited location.	Natural numbers.

The visitors file contains other information that may be relevant for the analysis. The distances between the centers of the geographical units were calculated according to the road network by a programmable interface (API) provided by Google Maps, considering the distance traveled between the two points using the road network. Moreover, as 2014 was the year of the FIFA World Cup in Brazil, some games could disturb the mobility. Information about the days, and holydays seasons are also included in the visitors’ file.

The second file is the OD matrix estimation, computed using two successive phone calls defining a trip. In this case, the origin may be other than the domicile, and each user can make more than one trip per day. The distribution of the call is very asymmetric, such that the necessity of two successive calls dramatically reduces the number of available records and only one OD pair per day could be estimated. The number of trips is adjusted to the population as described in Section 3. This file contains 1,019,667 records in tab-delimited format in the FGV Dataverse^6^. Table 2 presents the data dictionary.

**Table 2:** Data dictionary of the OD file.

Variable	Type	Description	Domain
day	String (10)	Date of the trip, starting at 2013-12-31.	363 dates
origin	String (20)	Location name where the trip was originated.	Name of 54 geographic units
destination	String (20)	Location name where the trip was destinated.	Name of 54 geographic units
travel_count_no_factor	Long integer	Number of trips computed from the raw data.	Natural numbers.
travel_count_fix_factor	Long integer	Number of trips computed with the fixed expansion factor.	Natural numbers.
travel_count_adap_factor	Long integer	Number of trips computed with the adaptive expansion factor.	Natural numbers.

### 3.2 Socioeconomic dataset

**Fig. 2:**
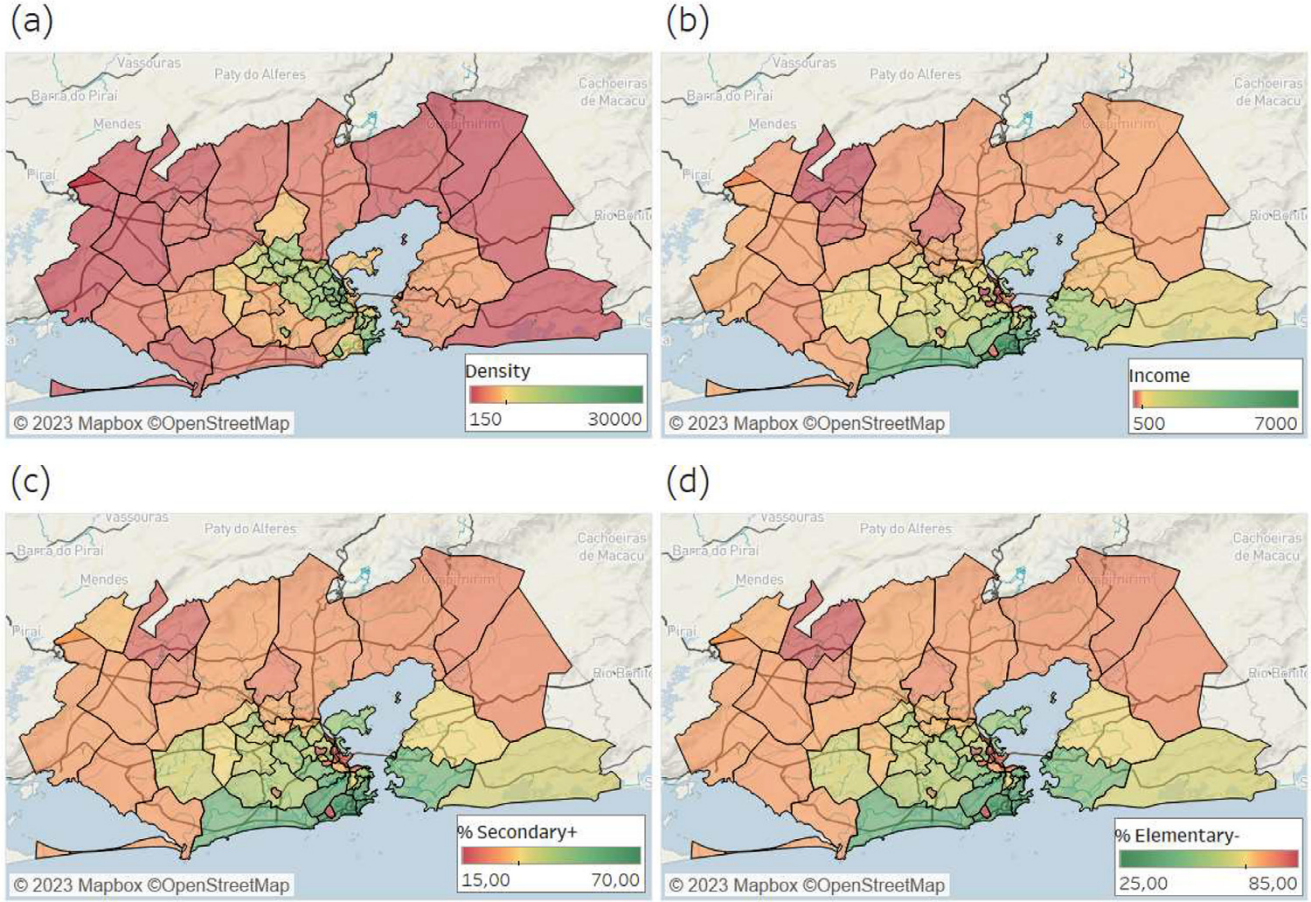
An example of socioeconomic data for the region: (a) population density; (b) average income (R$); (c) percentage of residents with secondary education, or higher; (d) percentage of residents with elementary school, or less.

The socioeconomic dataset in one file with an extract of the 2010 Brazilian census, provided by the Brazilian Institute for Geography and Statistics (IBGE). The file contains 55 socioeconomic variables for each geographic unit. These variables are often used for socioeconomic indicators such as sex, race, income, education, access to sanitation and clean water, etc. Any other variable or survey available in the IBGE spatial partitioning of the study area can be directly linked to the mobility files through the IBGE identification codes. The socioeconomic file is in tab-delimited format in the FGV Dataverse^7^. Table 3 presents the data dictionary including the IBGE identification code for each location, allowing linking to other databases. The IBGE location code is a sequence of numeric characters, where values with 7 numbers refers to a municipality, and 11 refers to a sub-district. An example of a visualization of socioeconomic variables is shown in Fig. 2.

**Table 3:** Data dictionary of socioeconomic file.

Subject/Variable	Type	Description	Domain
census_code	String (11)	IBGE identification code	Code of the 54 geographic units.
loc_name	String (20)	Location name.	Name of 54 geographic units
Education (12 indicators)	Integer	Education indicators.	Natural numbers.
Health (12 indicators)	Integer	Health indicators.	Natural numbers.
Housing (11 indicators)	Integer or float	Housing indicators.	Natural or Real numbers.
Work (7 indicators)	Integer or float	Work indicators.	Natural or Real numbers.
Mobility (13 indicators)	Float	Mobility indicators.	Real numbers.

### 3.3 Auxiliary files

The auxiliary files contain GIS shapefiles of the study area and data from the survey performed for the 2013 Urban Transport Plan for the Rio de Janeiro Metropolitan Area (PDTU) used for validation of OD estimation. Three files are available in this dataset:

• **PDTU file**^8^: This file contains commuting aggregated data gathered through the official survey for the 2013 Urban Transport Plan for the Rio de Janeiro Metropolitan Area (PDTU). The Urban Transport Master Plan conducted interviews with 9578 individuals residing in 4437 households. These individuals reported making varying numbers of trips per day, ranging from none to multiple trips, totaling 19,593 trips. During the interviews, participants were generally queried about the trip's starting point, destination, purpose, and the mode of transportation used. Each record contains the name of the OD pair and the number of trips. The PDTU file is in tab-delimited format.
• **RJMA Geographic shapefile**^9^: This shapefile contains the geographic borders of all locations in the study area, such as the sub-districts of Rio de Janeiro city, other municipalities of RJMA, and PDTU's traffic macro-zones. The primary key is the IBGE identification code for each location.
• **RJMA Voronoi shapefile**^10^**:** This shapefile contains the approximation of the geographic borders of all locations in the study area as an aggregation of Voronoi polygons related to tower position inside each location. The primary key is the IBGE code for each location.

**Fig. 3:**
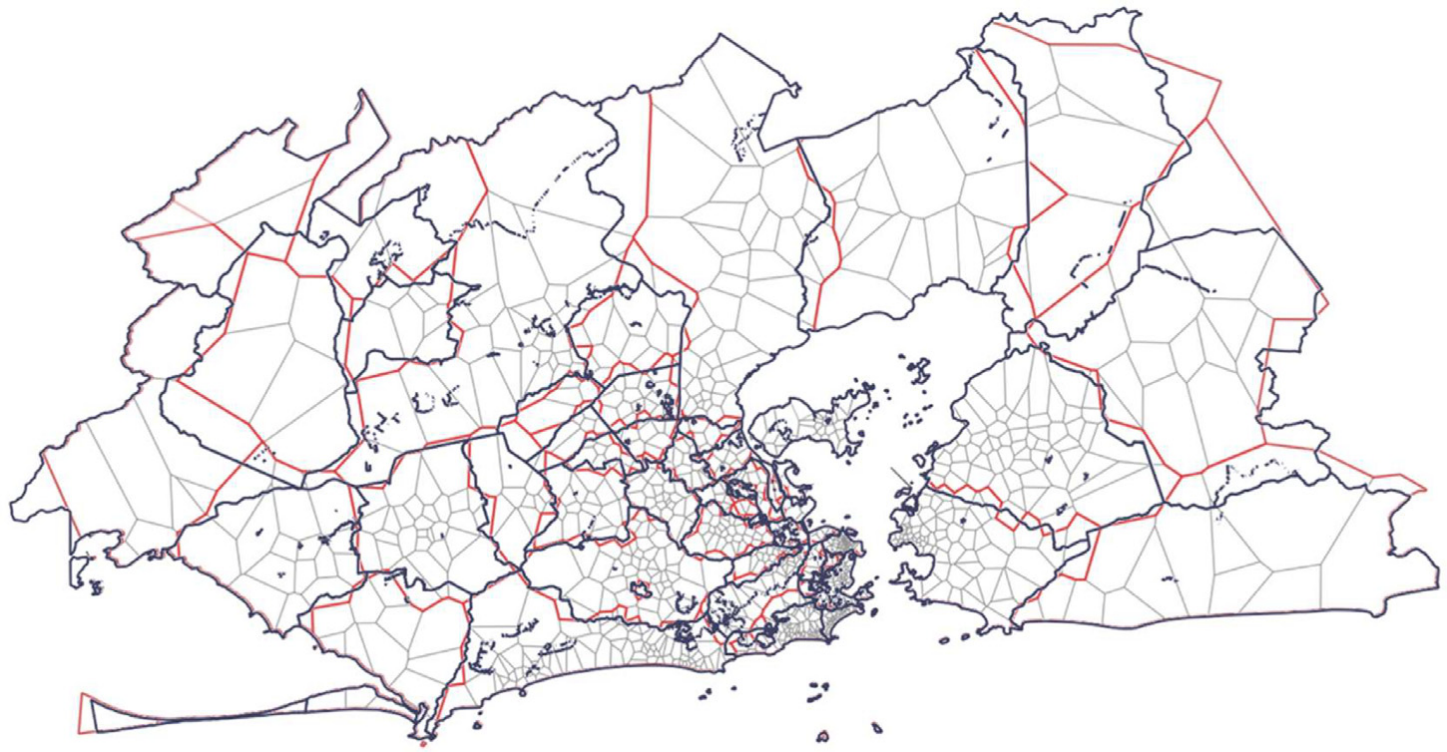
Superposition of the geographic borders of geographic units (in blue) and their approximation by aggregation (in red) of Voronoi polygons (in gray) in the RJMA. (For interpretation of the references to color in this figure legend, the reader is referred to the web version of this article.)

Fig. 3 presents the superposition of different shapefile layers. The Voronoi polygons (in grey) and their aggregation (in red) for each geographic unit are contained in the Voronoi shapefile. The geographic borders in the Geographic shapefile are shown in blue. The geographic borders are well approximated by the aggregation of Voronoi polygons, especially in denser areas where the coverage area of each tower is smaller.

## 4 Materials and Methods

Data collection for urban transport planning is costly and requires a long period of planning and execution and a limited sample size [5,6]. Call Detail Records (CDR) data is generated in large amounts by telephone operators and contains, among other information, the approximate location of the telephone call and the date and time of that activity. Despite some biases, such as penetration rate differences across the region, and the limitations in identifying the modal choice and the chosen routes, the use of CDR allows the identification of the main displacements of the population [2,[7], [8], [9], [10]].

The raw CDR data used to derive the data presented in this descriptor covers 363 days from December 31, 2013, to January 1, 2015 (4 days were missing), totaling 2.1 billion records of call made by for 2.9 million mobile phone users. Only outgoing voice call data was available for the study, so the dataset does not contain additional information, such as incoming calls and text messages (SMS), which implies less detailed monitoring of the users’ position. The information from each call record in raw CDR is shown in Table 4, where Cell ID was then substituted by the corresponding geographic coordinates (Latitude and Longitude) of the antenna's tower. The data were cleaned to avoid outliers, such that users with more than 100 calls per day or less than 10 calls per year were removed.

**Table 4:** Raw CDR data used in this study.

Item	Description
Day	Day of the record
Hour	Time of the record
Duration	Duration of the call
Code origin	The area code of the originating station of the call
ID origin	The encrypted ID of the originating source of the call
Code destination	area code of the destination station
ID destination	The encrypted ID of the destination of the call
Cell ID (origin)	The antenna code that processed the call at the source
Traffic	Type of traffic: international roaming, SMS, etc.
Hold_orig	The name of the carrier that processed the call at the source
Hold_dest	Name of the carrier that processed the call at the destination

The presumed residence of each user was computed as the most visited geographic unit between 8:00 p.m. and 6:00 a.m. of the next day during workdays and the entire day on Sundays and holidays. It is also required that the user be regularly detected in this location (at least five times) and that the number of visits at the most frequented location is always greater than the number of visits at the second most frequented location. The final dataset contains only users with an identified residence and counts 2.5 million users. It is considered that each user id in the raw CDR data corresponds to one person. The presumed domicile matches the 2010 population estimated by the IBGE census with a Pearson correlation coefficient of 90% [2].

In the visitors’ file, each day was divided into four shifts of 6 hours each, starting from 4:00 a.m., which is the time of less activity. The four shifts are: Morning, from 4:00 a.m. to 10:00 a.m.; Work, from 10:00 a.m. to 4:00 p.m.; Afternoon, from 4:00 p.m. to 10:00 p.m. and Night, from 10:00 p.m. to 4:00 a.m. of the next day. The number of visitors in each location was computed directly by the most frequent location visited by each user on each shift for each day.

The number of visitors estimated from the raw CDR dataset represents only the active users, such that it must be rescaled to represent all the population. There are different ways to rescale the estimation of the estimation. The simplest way is to consider a fixed market share and then calculate the scale factor by the ratio between the population and the number of users residing in the origin location. In this approach, the users are considered a sample of the population at origin for the calculation of the number of trips of the population. This approach is used to estimate the number of visitors in each location.

The number of visitors in each location is grouped according to the residence location, such that for each one there is the number of visitors from all the other locations, including the visited location, indicating people at home in that shift.

The algorithm used for the OD matrix estimation is based on similar approaches described in the literature [1,3,9]. Two successive calls in different cells are used to define a trip considering the first call location as origin and the second call location as destination. This method captures the actual commuting, but it is possible that the calls were made in transit and the actual commuting occurred between different locations. Additional parameters are used to avoid noise. It is required that the distance between origin and destination cells is at least $L_{min},$ and that the trip is performed within a time interval $T_{min}\leq \Delta T\leq T_{max}$. The parameters for the trip detection were $L_{min}=2\text{km}$ and 30min≤ΔT≤4h [2,4].

The algorithm of OD matrix estimation is shown in Algorithm 1. The scaling factor ($k_{i}$ in line 07) is computed fixed market share as in the visitors’ file. The fixed market share approach is a first approximation, but it does not consider the fluctuation in the number of users detected each day, which can be very different, for example, on weekdays and holidays. The adaptive scale factor is calculated for each day, considering the ratio between the number of detected users and the population at the domicile location. The number of trips in the OD file presents both estimations, using fixed and adaptive scaling factor.

**Algorithm 1:** OD matrix estimation from CDR data.

**INPUT**:	Call log database, the constants ΔT, and $L_{min}$, and table of distances between geographic units$T_{min}T_{mmax}$
**OUTPUT**:	OD Matrix

01	**Begin**
02	OD = NULL
03	**For** each day of the database
04	** For** each trip detected by two successive records of the same user, with distance $l_{ij}$ and in the time interval ΔT
05	** If**$l_{ij}>\;L_{min}$ and $T_{min}\leq \Delta T\leq T_{max}$
06	** **Identify presumed domicile i
07	** **Compute de expansion factor $k_{i}$
08	** **$OD_{ij}\leftarrow OD_{ij}$ + $k_{i}$
09	** End If**
10	** End For**
11	**End For**
12	Returns OD Matrix
13	**End**

The OD matrix was validated by comparing the number of trips with the published results in the 2013 Urban Transport Plan for the Rio de Janeiro Metropolitan Area (PDTU) [2]. The PDTU is based on a survey of individuals about their mobility behavior. This survey includes residence, occupation, origin, and destination of frequent trips, motivation, the mode of transport, among others. Only information on the origin and destination of frequent trips were used for comparison. The PDTU divides the RJMA into traffic zones based on transportation principles. Traffic zones are the same size as (but not equal to) census tracks and are aggregated in traffic macro zones. The macro zones define a spatial partitioning compatible with the one considered in this study by considering districts (aggregation of sub-districts) inside Rio de Janeiro city and groups of municipalities in the RJMA, outside Rio de Janeiro. The comparison of the OD estimation with PDTU OD is a good match, mainly for traffic macro zones [2].

## Ethics Statements

All private information was encrypted in the raw CDR data used to generate the datasets presented in this descriptor. Users' privacy has been preserved and comply with the confidentiality agreement signed by the authors. The derived data, described and shared here, do not identify any natural person.

## CRediT authorship contribution statement

**Júlio César Chaves:** Conceptualization, Methodology, Software, Data curation, Writing – original draft, Validation, Resources, Visualization. **Moacyr A.H.B. da Silva:** Conceptualization, Methodology, Funding acquisition, Validation, Formal analysis, Investigation, Writing – review & editing, Project administration. **Ricardo de Souza Alencar:** Validation, Formal analysis, Writing – review & editing. **Alexandre G. Evsukoff:** Conceptualization, Methodology, Data curation, Funding acquisition, Writing – original draft, Validation, Formal analysis, Resources, Writing – review & editing, Supervision, Project administration. **Vinícius da Fonseca Vieira:** Investigation, Validation, Writing – review & editing.

## Data Availability

FGV Dataverse (Original data).

## Footnotes

8 https://dataverse.fgv.br/dataset.xhtml?persistentId=hdl:10438.3/FK2/TYPWCM.

9 https://dataverse.fgv.br/dataset.xhtml?persistentId=hdl:10438.3/FK2/ALDYFQ.

10 https://dataverse.fgv.br/dataset.xhtml?persistentId=hdl:10438.3/FK2/N78E9A.

## Footnotes

4 In 2018, the RJMA was enlarged to include all municipalities of area code 21 and the municipality of Petropolis.

5 https://dataverse.fgv.br/dataset.xhtml?persistentId=hdl:10438.3/FK2/6AA6UL.

6 https://dataverse.fgv.br/dataset.xhtml?persistentId=hdl:10438.3/FK2/S5ECCO.

7 https://dataverse.fgv.br/dataset.xhtml?persistentId=hdl:10438.3/FK2/ALDYFQ.
